# Longitudinal High-Throughput Sequencing of the T-Cell Receptor Repertoire Reveals Dynamic Change and Prognostic Significance of Peripheral Blood TCR Diversity in Metastatic Colorectal Cancer During Chemotherapy

**DOI:** 10.3389/fimmu.2021.743448

**Published:** 2022-01-12

**Authors:** Yi-Tung Chen, Hung-Chih Hsu, Yun-Shien Lee, Hsuan Liu, Bertrand Chin-Ming Tan, Chia-Yin Chin, Ian Yi-Feng Chang, Chia-Yu Yang

**Affiliations:** ^1^ Molecular Medicine Research Center, Chang Gung University, Taoyuan, Taiwan; ^2^ Research Center for Emerging Viral Infections, Chang Gung University, Taoyuan, Taiwan; ^3^ Division of Hematology-Oncology, Chang Gung Memorial Hospital at Linkou, Tao-Yuan, Taiwan; ^4^ College of Medicine, Chang Gung University, Tao-Yuan, Taiwan; ^5^ Department of Biotechnology, Ming Chuan University, Taoyuan, Taiwan; ^6^ Department of Cell and Molecular Biology, College of Medicine, Chang Gung University, Taoyuan, Taiwan; ^7^ Graduate Institute of Biomedical Sciences, College of Medicine, Chang Gung University, Taoyuan, Taiwan; ^8^ Division of Colon and Rectal Surgery, Chang Gung Memorial Hospital, Taoyuan, Taiwan; ^9^ Department of Biomedical Sciences, College of Medicine, Chang Gung University, Taoyuan, Taiwan; ^10^ Department of Neurosurgery, Lin-Kou Medical Center, Chang Gung Memorial Hospital, Taoyuan, Taiwan; ^11^ Department of Microbiology and Immunology, College of Medicine, Chang Gung University, Taoyuan, Taiwan; ^12^ Department of Otolaryngology-Head and Neck Surgery, Chang Gung Memorial Hospital, Taoyuan, Taiwan

**Keywords:** T cell repertoire, metastatic colorectal cancer, prognosis, peripheral blood, chemotherapy

## Abstract

Colorectal cancer (CRC) is a major cause of cancer mortality and morbidity. Despite advances in chemotherapy and targeted therapy, unsustainable clinical benefit was noted due to recurrence and therapy resistance. The immune status of the cancer patient may affect the effectiveness of disease treatments. The dynamic change in the T-cell receptor (TCR) repertoire might be a clinical parameter for monitoring treatment responses. In this study, we aimed to determine the characteristics and clinical significance of the TCR repertoire in patients with unresectable metastatic colorectal cancer (mCRC). Herein, we comprehensively profile 103 peripheral blood samples from 20 healthy controls and 16 CRC patients with a follow-up of 98 to 452 days to identify hypervariable rearrangements of the TCRα and TCRβ repertoires using high-throughput sequencing. We found that TCRα repertoires, TCRβ repertoires, and CDR3 clonotypes were altered in mCRC patients compared with healthy controls. The diversity of TCR repertoires and CDR3 clonotypes decreased in most mCRC patients after therapy. Furthermore, compared with baseline TCR diversity, patients whose TCR diversity dropped considerably during therapy had better treatment responses, including lower CEA and CA19-9 levels and smaller tumor sizes. TCR baseline diversity was also significantly associated with partial response (PR) status (odds ratio: 5.29, *p* = 0.04). In conclusion, the present study demonstrated the association between dynamic changes in TCR diversity during chemotherapy and clinical outcomes as well as the potential utility of the TCR repertoire in predicting the prognosis of cancer treatment.

## Introduction

Colorectal cancer (CRC) is one of the most common cancers worldwide. It remains a major cause of cancer mortality and morbidity among men and women globally (1, 2). For most surgical CRC patients, tumor recurrence or metastasis is still a major challenge; most recurrences occur within 2 years of surgery, and 90% by 5 years. Advances in active agents and biomarker-driven treatment selection have improved outcomes in metastatic CRC patients. However, unsustainable clinical benefit was noted, and recurrence and therapy resistance are emerging as the major causes for limited clinical improvement. Earlier detection of tumor recurrence and monitoring of therapy outcome may give physicians more time for disease management. Imaging technology, including computed tomography (CT) or magnetic resonance imaging (MRI), can be used to measure the tumor size in follow-up patients. However, these examinations cannot be performed frequently due to radiation side effects (3). Serum carcinoembryonic antigen (CEA) and carbohydrate cell surface antigen 19-9 (CA19-9) are recommended as tumor markers in CRC for tumor detection and monitoring of treatment responses. However, their sensitivity and specificity issues limit their clinical usefulness (4–6). Thus, biomarker studies include early detection of therapy resistance, and ensuring that patients are exposed to as many active therapies as possible is necessary.

Recent years have brought a large number of approvals of immunomodulatory therapies for cancer treatment. T lymphocytes are key immune cells in adaptive immunity and are responsible for the antitumor immune response *via* specific receptor-mediated recognition of tumor-associated antigens. The T-cell receptor (TCR) repertoire then refers to the sum of T cells with functional diversity in the immune system of an individual person at any given time. Most TCRs consist of α chains and β chains, while the rest contain γ chains and δ chains. The TCR repertoire can be thought of as a mirror of human immune status, and its dynamic change could be a promising biomarker to monitor immunomodulatory therapies (7). Therefore, profiling the immune repertoire characteristics of T cells under certain physiological and pathological conditions provides context-dependent information on the clone distribution and function of T cells (8–10). The specificity and diversity of TCRs are predominantly derived from the highly variable complementarity determining region 3 (CDR3) and random rearrangement and junction region mutation of V(D)J regions (11). Several studies have demonstrated that TCR and CDR3 diversity are important in cancer diagnosis, therapy, and prognosis (12–17). Performing noninvasive peripheral blood repertoire profiling provides information for monitoring the TCR repertoire in cancer patients for comparison purposes (18). Monitoring specific T-cell clonal distribution in response to treatment could help to illustrate whether the treatment is effective, serving as an important reference value for evaluation of treatment efficacy and determining recurrence, metastasis and prognosis.

Although some studies describe the oncological significance of TCR repertoire diversity in CRC, only CDR3 spectratypes are analyzed (19–21). Comprehensive profiling studies of the TCR repertoire and a longitudinal cohort for monitoring TCRα and TCRβ repertoire dynamics during therapy and elucidating the functional consequences of T cells in cancer progression and treatment are still lacking. In the present study, we used high-throughput sequencing to comprehensively profile the TCRα and TCRβ repertoires in patients with unresectable metastatic CRC during therapy. Our results aimed to provide the clinical insight for monitoring therapeutic response within a non-invasive approach, and which demonstrated the association of TCR diversity and clinical outcomes as well as the potential utility of TCR repertoires in cancer prognosis.

## Materials and Methods

### Study Cohort

A total of 36 subjects, including 20 healthy controls and 16 metastatic CRC patients, were enrolled in this study. The study was approved by the Institutional Review Board at Chang Gung Memorial Hospital, Taiwan (IRB 201601848B0 and IRB 201801576B0). Prior to sample collection, written informed consent was obtained for all subjects. Peripheral blood samples were obtained from 20 age-matched healthy controls (62.6 ± 10.48 years old) and 16 CRC patients (62.38 ± 12.62 years old) before therapy. Among the 16 CRC patients, 67 peripheral blood samples were collected from 13 patients with follow-up every two months for approximately 98 to 452 days. We analyzed a total of 103 samples from these subjects. Clinical information, including demographic information, clinical characteristics, and histopathological features, was collected from medical records, and the therapeutic response was evaluated by imaging according to the Response Evaluation Criteria in Solid Tumors (RECIST). Survival was also retrospectively analyzed for all evaluable patients.

### Isolation of Mononuclear Cells and RNA Extraction

Fresh peripheral blood from healthy controls and CRC patients was collected in EDTA-treated Vacutainer tubes (BD Biosciences, Franklin Lakes, NJ). Peripheral blood mononuclear cells (PBMCs) were isolated following the standard procedure, and total RNA from PBMCs was extracted using TRIzol reagent (Invitrogen, Carlsbad, CA) according to the manufacturer’s protocol. The quality and quantity of purified RNA were confirmed using the Agilent 2100 Bioanalyzer (Agilent Technologies, Santa Clara, CA).

### Multiplex PCR Amplification of the TCRα and TCRβ Repertoires and High-Throughput Sequencing

A multiplex PCR amplification reaction was used to amplify the TCR immune repertoire. Human TCRα and TCRβ libraries were prepared using the HTAI-M and HTBI-M Kits (iRepertoire, Inc.) according to the manufacturer’s instructions and sequenced on the Illumina MiSeq platform. Briefly, RNA samples were reverse transcribed into cDNA with adaptor-specific primers. During the first round of PCR, nested gene-specific primers targeting each of the V and J (or C) genes were used. The first PCR program consisted of 40 min at 50°C and 15 min at 95°C, followed by 10 cycles of denaturation at 94°C (30 seconds), annealing at 60°C (5 min), and extension at 72°C (30 seconds), followed by 10 cycles at 94°C (30 seconds) and 72°C (3 min), with a final extension at 72°C for 5 min. The PCR products were purified using AMPure XP beads (Beckman Coulter Life Sciences), followed by second-round PCR. Second-round PCR was carried out with universal and specific barcode primers, with reaction conditions consisting of a 15 min denaturation at 95°C, followed by 30 cycles of denaturation at 94°C (30 seconds), annealing at 72°C (90 seconds), and a final extension at 72°C for 5 min. The amplicons were purified from the 6% TBE-PAGE gel by size selection for 400-500 bp. The yield and size distribution for libraries were assessed using the Agilent 2100 Bioanalyzer using the High Sensitivity DNA Assay (Agilent Technologies). Equal concentrations of each library with different barcodes were pooled and sequenced using the Illumina Paired-End Sequencing Kit (MiSeq, Illumina). The sequencing reads were assessed for quality, processed for barcode sequencing decoding, and aligned in the following section.

### Bioinformatics and Statistical Analyses for the Immune Receptor Repertoire

Sequencing data in fastq format were uploaded to the iRepertoire webserver (https://irweb.irepertoire.com/ir/index) for V(D)J recombination alignment in the TCRα and TCRβ chains (22). Complete mapped sequencing data were transferred into the VDJtools algorithm (https://vdjtools-doc.readthedocs.io/en/master/index.html) for advanced analyses (23), including clonal numbers/types, rarefaction plots, repertoire richness/evenness (Chao1/Shannon index), unique CDR3 species and frequency. All statistical analyses were performed using Student’s *t*-test. P values < 0.05 were considered significant (*p < 0.05, **p < 0.01, ***p < 0.001). For the follow-up subjects, weekly intervals were used for differentiating samples, and the nearby recognized sample was selected for relevant analyses. Pearson coefficient analysis and Jaccard algorithms for sample similarity and clonal distribution were conducted using MATLAB arithmetic operations.

## Results

### Clinical Characteristics of the Study Population

**Figure 1:**
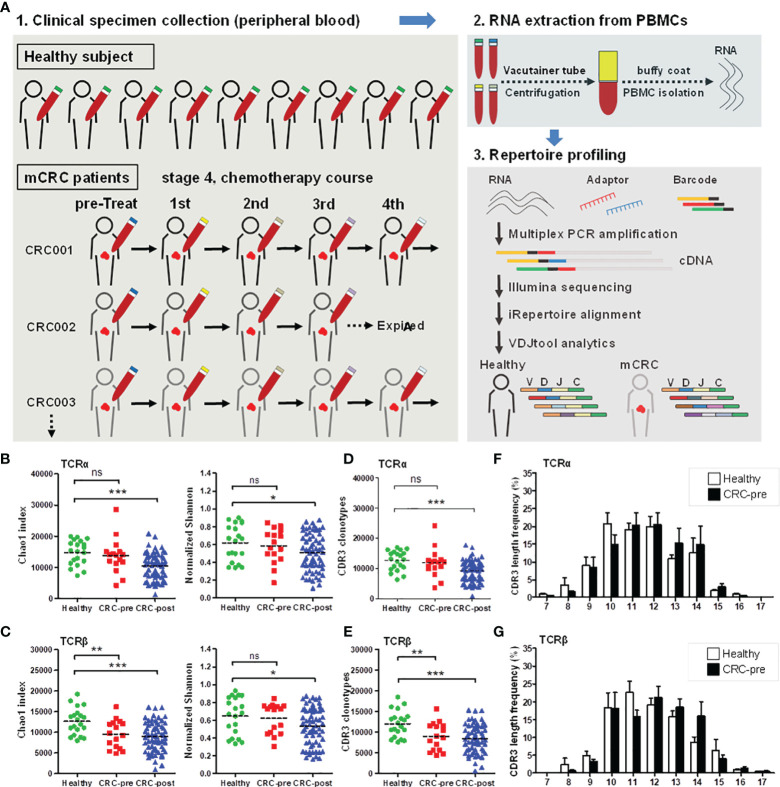
TCRα and TCRβ repertoires and CDR3 length distribution in CRC patients and healthy controls. **(A)** Schematic representation of the current experimental design. **(B**, **C)** The Chao1 and Shannon index of TCRα **(B)** and TCRβ **(C)** repertoires in healthy controls (Healthy; green circles). CRC patients before therapy (CRC-pre; red squares), and follow-up patients with therapy (CRC-post; blue triangles) were sequenced and analyzed using VDJtools software, and the results are presented as the mean ± SEM in the column scatterplot. **(D**, **E)** TCRα **(D)** and TCRβ **(E)** CDR3 clonotypes from healthy, CRC-pre, and CRC-post repertoires were determined using VDJtools analysis, and the number of CDR3 clonotypes in subjects is shown as a column scatterplot. **(F**, **G)** CDR3 coding length and frequency in healthy (white columns) and CRC-pre (black columns) TCRα **(F)** and TCRβ **(G)** repertoires were obtained using VDJtools analysis, and the amino acid length spectrum (x-axis) and the corresponding frequency (y-axis) are depicted in bar graphs. Statistical significance was determined using Student’s t-test. ns p > 0.05, *p < 0.05, **p < 0.01, ***p < 0.001.

We analyzed 103 peripheral blood samples from 16 metastatic CRC patients and 20 age-matched healthy controls. The healthy volunteers who were reported with no polyps or cancerous tissues by colonoscopic findings were enrolled as healthy controls in our study. Among these 16 CRC patients, 13 patients with available posttherapy samples every two months for follow-up for approximately 452 days were identified in the follow-up group; and the study design is illustrated as in **Figure 1A**. The clinical characteristics, including CEA and CA19-9 levels, tumor size, treatment response, and white blood cell counts, of the enrolled subjects are listed in **Table 1** and **Supplementary Table S1**. The average age of the 16 CRC patients was 62.38 years. The majority of 16 CRC patients were male (68.75%), had left side colon cancer (81.25%), and had a synchronous metastasis pattern (81.25%). A similar distribution of clinical parameters was present in the follow-up cohort. Initial anticancer treatment included FOLFIRI (Folinic acid, 5-FU, IRInotecan) chemotherapy and targeted therapy with bevacizumab or cetuximab. Among the 13 follow-up CRC patients, 46% were treated with bevacizumab/FOLFIRI, and 54% were treated with cetuximab/FOLFIRI.

**Table 1:** Characteristics of subjects in healthy and colon cancer patients.

Characteristics	Healthy control	Colorectal cancer	Follow-up
**Number of patients**	20	16	13
**Age (years)**			
range	53 - 74	42-80	47-78
mean ± SD	62.6 ± 10.48	62.38 ± 12.62	65.00 ± 11.42
**Gender**			
Male	9	11	8
Female	11	5	5
**Metastases**			
synchronous	–	13	10
metachronous	–	3	3
**Colorectal tumor location**			
Right side	–	3	1
Left side	–	13	12
**First line therapy**			
Bevacizumab/FOLFIRI	–	6	6
Cetuximab/FOLFIRI	–	8	7
FOLFIRI	–	2	0
**Microsatellite Stablilty**			
Microsatellite Stable (MSS)	–	13	–
Unknown	–	3	–

### TCRα and TCRβ Repertoire Diversity in CRC Patients and Healthy Controls

To characterize the TCRα and TCRβ repertoire dynamics in CRC patients, peripheral blood specimens were collected from 16 CRC treatment-naïve patients (CRC-pre; N = 16), 13 follow-up CRC patients (CRC-post; N = 67), and 20 healthy controls (N = 20). A total of 103 RNA samples were isolated from PBMCs and subjected to multiplex PCR technology for semiquantitative amplification of cDNA with specific nested primers, and first-round PCR was performed with specific primers for the V, D, and J genes (iRepertoire, Inc.). Then, the second round of PCR was performed using unique barcoded primers. The multiplex amplified libraries were pooled equally and sequenced on the Illumina MiSeq platform with 2 × 250 paired-end v3 sequencing reagents (Illumina, USA). Sequencing reads were processed using bioinformatics analysis for TCR repertoire profiling. The average number of TCRα paired reads per sample was 216,194 ± 26,491 for healthy controls, 278,350 ± 28,457 for CRC-pre patients, and 235,999 ± 15,188 for follow-up CRC-post patients. The average number of TCRβ paired reads per sample was 337,664 ± 30,612 for healthy controls, 356,382 ± 37,149 for CRC-pre patients, and 363,006 ± 18,627 for follow-up CRC-post patients. Sequencing read counts were comparable between groups (**Supplementary Figure S1A**), and sample-based rarefaction analyses suggested an adequate sequencing depth for the TCR repertoire profiling in each group, including healthy controls (**Supplementary Figure S1B**), the CRC-pre group (**Supplementary Figure S1C**), and the CRC-post group (**Supplementary Figure S1D**). The richness (Shannon index) of the TCRα and TCRβ repertoires was inversely correlated with age in healthy controls (correlation coefficients, r = -0.38 and -0.63 for TCRα and TCRβ, respectively; **Supplementary Figure S2A**), which is consistent with the decreased TCR repertoire diversity in elderly people (24, 25). To control for the influence of age on TCR repertoire diversity, age was matched between healthy controls and CRC patients. In CRC-pre patients, there was no correlation between age and TCRα or TCRβ repertoire diversity, suggesting a disturbed TCR repertoire in cancer patients (26) (**Supplementary Figure S2B**). Next, we compared the TCR diversity between different groups. The Chao1 and Shannon indexes were similar between healthy controls and CRC-pre patients in the TCRα repertoire (**Figure 1B**). In the analysis of the TCRβ repertoire, the Chao1 index was significantly decreased in CRC-pre patients compared to healthy controls (**Figure 1C**, left panel), whereas the Shannon index was similar between healthy controls and CRC-pre patients (**Figure 1C**, right panel). Interestingly, the Chao1 and Shannon indexes were significantly decreased in CRC-post patients compared with healthy controls in both the TCRα and TCRβ repertoires (**Figures 1B, C**).

### Professional CDR3 Clonotypes in CRC Patients and Healthy Controls

The CDR3 region is responsible for TCR recognition of highly variably antigens (27, 28). In the TCRα and TCRβ repertoires, the CDR3 clonotypes were positively correlated with the Chao 1 index (r = 0.998, p = 0.0001) (**Supplementary Figure S3**). In the TCRα repertoires, the CDR3 clonotypes were similar between CRC-pre patients and healthy controls; however, they were significantly decreased in CRC-post patients (**Figure 1D**). In addition, reduced TCRβ CDR3 clonotypes showed a decrease in Chao1 diversity in CRC-pre and CRC-post patients compared to healthy controls (**Figure 1E**), and accordingly, suggested differentially expressed CDR3 assemblies in healthy controls and CRC patients. These results suggest that the TCR repertoire profile changed during the course of chemotherapy treatments.

**Figure 2:**
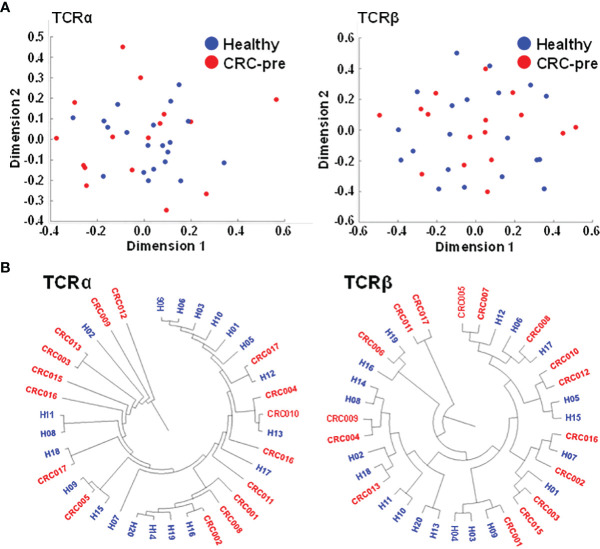
Multidimensional scaling and phylogenetic analyses showing the similarities in CDR3 clonotypes of TCR repertoires. **(A)** TCRα and TCRβ CDR3 clonotypes of healthy controls (n = 20; blue circles) and CRC-pre samples (n = 16; red circles) were subjected to multidimensional scaling to analyze the similarities in CDR3 sequences across samples. **(B)** Sample-based diversity matrices of CDR3 clonotypes in healthy controls and CRC-pre samples were analyzed using VDJtools to illustrate the distances between samples. The results are represented as a hierarchical phylogenetic tree.

CDR3 coding frequency analysis showed the approximate peptide length (ten to fourteen amino acids) for the CDR3 region in both healthy controls and CRC-pre patients (**Figures 1F, G**), and no biased CDR3 encoding pattern was uncovered between groups. Multidimensional scaling analysis based on TCRα or TCRβ CDR3 clonotypes showed differing expression profiles of individual samples (**Figure 2A**), which showed the development of the TCR repertoire as a long-term and multifactorial process. Furthermore, phylogenetic analysis of the CDR3 clonotype demonstrated that no exclusive TCRα and TCRβ repertoire clusters were observed for healthy controls and CRC-pre patients (**Figure 2B**). The results of the analysis of non-preferential usage for the TCR repertoire between healthy controls and CRC-pre patients revealed individual differences in the immune repertoire.

To investigate whether there were public TCRs that responded to CRC, we analyzed the shared immune repertoires that responded to CRC patients but not to healthy controls. Although no representative clonotypes for CRC were uncovered in all 16 CRC patients, uniquely expressed CDR3 clonotypes in at least 4 CRC-pre patients were still recognized in the TCRα and TCRβ repertoires **(** **Supplementary Tables S2, S3**). The 2 most prevalent CDR3 motifs (CAFPGAGSYQLTF, CAVVKAAGNKLTF) of the TCRα repertoire were detected in 6 out of 16 CRC-pre patients (37.5%). The 2 most prevalent CDR3 motifs (CASSGDSNQPQHF, CASSPPGSSYNEQFF) in the TCRβ repertoire were detected in 5 out of 16 CRC-pre patients (31.25%). On the other hand, there were various preferential CDR3 clonotypes found in at least 5 healthy controls but not in CRC patients (**Supplementary Tables S4, S5**), and the 2 most prevalent CDR3 motifs (CAYAGNNRKLIF and CAVPTQGGSEKLVF) in the TCRα repertoire were observed in 9 out of 20 healthy controls (45%).

### TCRα and TCRβ Repertoire Landscape in Healthy Controls and CRC Patients Before Therapy

**Figure 3:**
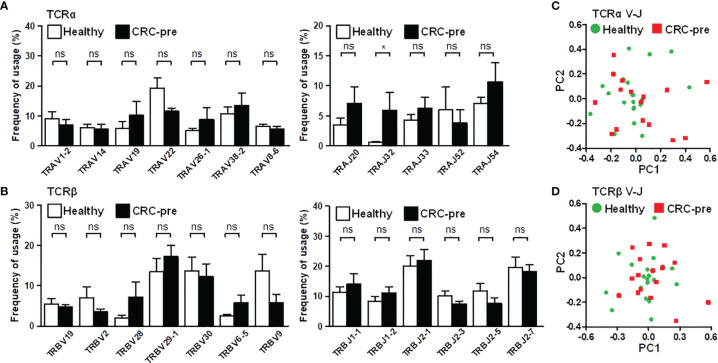
TCRα and TCRβ V and J gene usage in CRC patients and healthy controls. **(A)** Prevalent TCRα variable genes (TRAV; left panel) and joining genes (TRAJ, right panel) in healthy controls (white columns) and CRC-pre samples (black columns) are illustrated in bar graphs. **(B)** Highly expressed TCRβ variable (TRBV; left panel) and joining genes (TRBJ, right panel) in healthy controls (white columns) and CRC-pre samples (black columns) are shown as bar graphs. Statistical significance was determined using unpaired Student’s t-test method. Principal component analysis (PCA) of TCRα **(C)** and TCRβ **(D)** V-J gene recombination across healthy controls (n = 20; green circles) and CRC-pre samples (n = 16; red squares). ns p > 0.05, *p < 0.05.

Nucleotide addition and deletion in the CDR3 region showed the increased diversity of the CDR3 region caused by identical genetic recombination, which suggested divergently expressed CDR3 could be ascribed to similar variable (V) and joining (J) gene usage. Based on this hypothesis, we first assayed the frequency of prevalent V and J genes whose expression was higher than five percent. Most prevailing V/J gene expression was similar between healthy controls and cancer patients (**Figures 3A, B**). The J32 segment of TCRα (TRAJ32) was enhanced in CRC-pre patients compared to healthy controls (**Figure 3A**). In parallel with the results displayed in the bar graph, a heat map of public V and J gene usage showed no clearly expressed genetic signature between samples in different groups (**Supplementary Figure S4**), which revealed the individual specificity for immune receptors (29). Exact V and J gene usage for individual samples showed a complicated rearrangement in shaping repertoire diversity (**Supplementary Figure S5**), which showed the exclusive V and J gene usage in each person. Accordingly, the principal component analysis (PCA) for V-J recombination demonstrated the considerable difference in individual subjects, and healthy controls and CRC-pre patients were not distinguished by this clustering assay in the TCRα (**Figure 3C**) or TCRβ (**Figure 3D**) repertoires.

### Decreased TCR Repertoire Diversity Is Associated With Clinical Outcome

**Figure 4:**
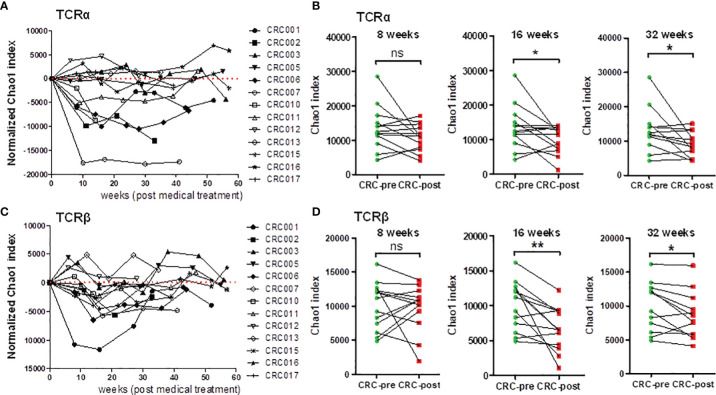
Dynamic analysis of TCRα and TCRβ repertoire diversity in follow-up CRC patients who underwent chemotherapy. **(A)** The normalized TCRα Chao1 index from a total of 80 samples, including 13 CRC-pre samples (CRC001 to CRC017) and 67 follow-up CRC-post samples, was used to analyze and normalize to the value of paired baseline CRC-pre samples. The normalized Chao1 index (y-axis) and sample collection time (x-axis; weeks) were used to plot the connected scatterplot. **(B)** TCRα Chao1 index at the indicated time points (8 weeks, 16 weeks, and 32 weeks) in follow-up CRC-post (red squares) subjects and paired CRC-pre (green circles) subjects were compared. **(C)** The normalized TCRβ Chao1 index from 80 samples was determined and analyzed as described in panel **(A)**. **(D)** The TCRβ Chao1 index results at the indicated time points (8 weeks, 16 weeks, and 32 weeks) in follow-up CRC-post (red squares) subjects and paired CRC-pre (green circles) subjects were compared. Statistical significance was determined using paired Student’s t-test. ns p > 0.05, *p < 0.05, **p < 0.01.

The diversity of the TCRα and TCRβ repertoires was significantly decreased in CRC patients after medical treatment, showing the association between TCR repertoire dynamics and chemotherapeutic treatment (**Figure 1**). In addition, individuals had unique TCR gene configurations, and no specific clustering was uncovered between healthy controls and CRC patients. Based on these observations, we alternatively profiled individual CRC patients’ TCR repertoires during their medical treatment, and this continuous collection approach can be used to identify substantial changes in the immune repertoire. The Chao1 index was used to estimate the dynamic change in TCR diversity in CRC patients during chemotherapy. In the TCRα repertoire, time course plotting for the Chao1 index showed a declining tendency after medical therapy in 9 out of 13 follow-up patients (**Figure 4A**). Furthermore, the Chao1 index was significantly decreased in CRC patients after 16 and 32 weeks of treatment compared with CRC-pre patients (**Figure 4B**). Similarly, 9 out of 13 patients had a decreased Chao1 index in the TCRβ repertoire after medical therapy (**Figure 4C**), and the Chao1 index was significantly decreased in CRC patients after 16 and 32 weeks of treatment compared with CRC-pre patients (**Figure 4D**). These results showed that significantly decreased TCR repertoire diversity was observed in approximately 69% (9 out of 13 patients) of CRC patients over the course of medical treatment.

**Figure 5:**
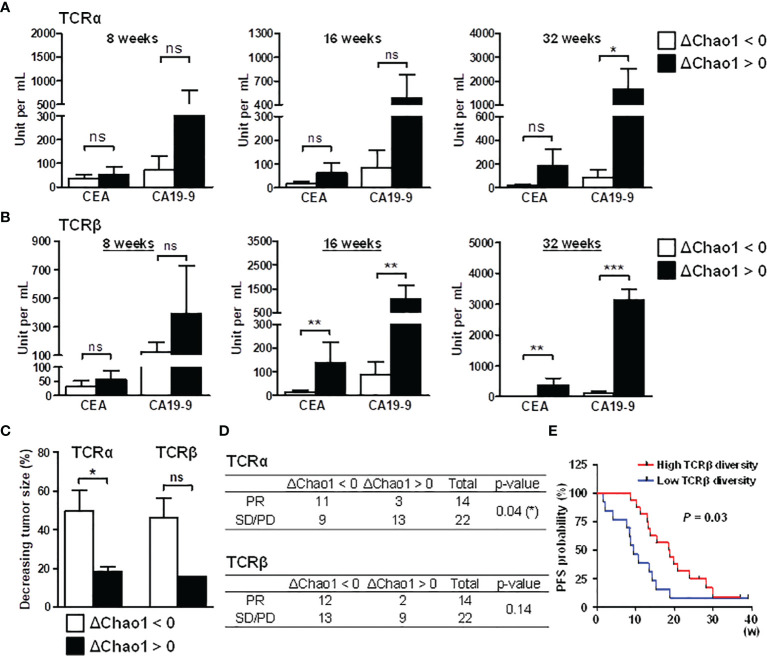
Association between decreased TCR repertoire diversity and prognosis in CRC patients. Compared to the baseline Chao1 index, patients with a decreased Chao1 index during chemotherapy were categorized into the diversity decreasing group (ΔChao1 < 0); patients with an increased Chao1 index during chemotherapy were considered the diversity increasing group (ΔChao1 > 0). To determine the association between TCR and diagnosis, TCRα **(A)** and TCRβ repertoire **(B)** diversity, the differences in CEA and CA19-9 levels were analyzed between the ΔChao1 > 0 and ΔChao1 < 0 groups at the indicated time points of 8 weeks, 16 weeks, and 32 weeks after therapy. Statistical significance was determined using Mann-Whitney U test method. **(C)** The association between the dynamics of the Chao1 index and tumor size was analyzed. The Chao1 deviation at 16 weeks after therapy compared to baseline diversity was categorized into diversity decreasing group (ΔChao1 < 0) or diversity increasing group (ΔChao1 > 0). The percentage of decreased tumor size at 16 weeks was calculated by normalizing to the corresponding tumor size of CRC-pre subjects. Statistical significance was determined using Mann-Whitney U test method. **(D)** The association between TCRα and TCRβ Chao1 deviation in treatment response, including partial response (PR), stable disease (SD) and progressive disease (PD), was analyzed using Pearson’s chi-square test. A statistical p-value lower than 0.05 was noted. **(E)** The association between baseline TCRβ diversity and PFS was assessed, and the obtained *P*-value is shown. ns p > 0.05, *p < 0.05, **p < 0.01, ***p < 0.001.

Based on the dynamic change in the Chao1 index during chemotherapy, we studied the correlation between patient TCR diversity and treatment outcome. The dynamic change in the Chao1 index was used for patient subgrouping. Compared to the baseline Chao1 index, patients with a decreased Chao1 index during chemotherapy were considered the diversity decreasing group (ΔChao1 < 0); patients with an increased Chao1 index during chemotherapy were considered the diversity increasing group (ΔChao1 > 0). The relevant clinical parameters, including CEA, CA19-9, tumor size, and white blood counts, in the diversity decreasing group (ΔChao1 < 0) and the diversity increasing group (ΔChao1 > 0) were compared. In the TCRα repertoire, there was a trend showing higher CEA and CA19-9 levels in the ΔChao1 > 0 group than the ΔChao1 < 0 group at 8, 16, and 32 weeks (**Figure 5A**). In the TCRβ repertoire, the phenomenon was observed at 16 and 32 weeks (**Figure 5B**). The correlation between CEA and CA19-9 levels and TCR diversity suggested the prognostic potential of immune repertoire profiles for monitoring CRC treatment responses. Analysis of other clinical parameters, including the number of eosinophils, and basophils, showed no significant difference between the ΔChao1 > 0 and ΔChao1 < 0 groups (**Supplementary Figures S6A, B**). In particular, the number of lymphocytes and monocytes in ΔChao1 > 0 group were higher at 16 weeks and 32 weeks after chemotherapy, respectively (**Supplementary Figure S6A**). In addition, sex was a contributive factor for the mortality of CRC (30); however, the nearly equivalent number of males and females in the Chao1 subgroup (data not shown) suggested that sex was not the contributing factor in the current study. Alternatively, the results revealed the significance of dynamic changes in TCR diversity during the therapeutic course, which was correlated with CEA and CA19-9 levels.

Due to the limited CT volumetric operation, the tumor size of each collection time point could not be appropriately received. Consequently, we focused on the third collection time point (average 16 weeks after treatment) where most patients received CT scanning for therapeutic effectiveness estimation. The tumor decrease rate was measured based on the tumor size before and after treatment at 16 weeks, and it represented the criterion for determining patients’ treatment response. In the TCRα repertoires, CRC patients in the ΔChao1 < 0 group showed a higher tumor decreasing rate than patients in the ΔChao1 > 0 group (**Figure 5C**). Similarly, TCRβ repertoires showed an association trend between TCR diversity and the tumor reduction rate (**Figure 5C**), although the sample sizes were limited and did not reach statistical significance (p = 0.07). In addition, chi-square analysis was performed to inspect the dynamic change in TCR diversity for treatment responses. The treatment responses of patients were evaluated by clinicians according to the clinical criteria of partial response (PR), stable disease (SD), or progressive disease (PD). In the TCRα repertoires, CRC patients in the ΔChao1 < 0 group had better treatment responses than those in the ΔChao1 > 0 group (p = 0.04) and odds ratio (5.29), which indicated a strong association between TCR diversity and treatment responses (**Figure 5D**). Furthermore, we included the additional data of 14 patients, with a total of 30 samples to assess the association of TCRβ diversity to progression-free survival (PFS), and which showed the patients with higher TCRβ diversity at baseline had the longer progression-free survival (PFS) interval than those with lower TCRβ diversity (**Figure 5E**). Furthermore, we applied the COX regression model to analyze the other potential risk factors serving as PFS biomarkers (31), including TCR diversity, tumor size, and CEA value. Cox regression analysis for PFS revealed a 0.84 hazard ratio of TCR diversity suggesting the 15.6% decreased hazard for each one-unit increase in the TCR diversity (**Supplementary Figure S6C**). Compared to CEA value and tumor size, measurement of TCR diversity of cancer patients may provide a better estimation for disease survival. This finding was consistent with the association between TCR diversity and tumor malignancy (32), and it revealed the significance of TCR repertoires in cancer treatment responses. In conclusion, these observations demonstrated the association between TCR diversity and clinical outcomes as well as the prognostic and predictive biomarkers of immune repertoires. These results implied that decreased TCR diversity during chemotherapy favors effective treatment responses in CRC patients.

### Longitudinal TCR Repertoire Profiling Reveals Immune Repertoire Stability and Individual-Specific Characteristics

**Figure 6:**
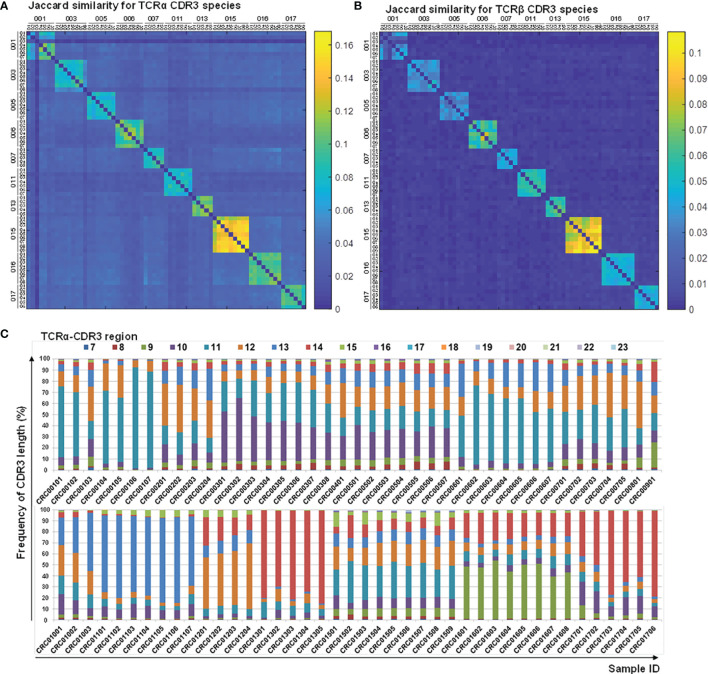
Characteristics of CDR3 sequences and length distribution before and after therapy. CDR3 sequences from the TCRα **(A)** and TCRβ **(B)** repertoires of follow-up CRC patients (CRC-pre and paired CRC-post) were subjected to Jaccard coefficient analyses to illustrate the similarity between samples, and the coefficient between any two given samples was colored coded and illustrated using a heat map. The coefficient scale is shown. **(C)** CDR3-encoded amino acid length distributions of the TCRα repertoires are presented as color-coded percentage bar graphs. The sample ID is the x-axis, and the peptide length frequency is the y-axis.

Correlation analyses on longitudinal subjects (CRC-pre and CRC-post) were performed to detect perceptible changes in TCR repertoires during medical treatments. The Jaccard index for CDR3 motifs showed a considerable difference between CRC patients (**Figures 6A, B**), which suggested a distinctive immune repertoire in each patient. Incidentally, the frequency distribution for CDR3 peptide length showed individual differences in CDR3 motifs (**Figure 6C** **;** **Supplementary Figure S7**). Pearson correlation analysis of TCRα and TCRβ V/J genes showed that a portion of the V/J gene configuration was present in some patients, but most patients had individual V/J gene usage (**Supplementary Figure S8**). This assay provided a reasonable explanation for the lack of public immune repertoires identified in CRC patients, which supported the unique gene recombination for repertoire diversity. These analyses demonstrated the divergence and distinction of TCR repertoires in individuals, which revealed the significance of repertoire sequencing in precision medicine.

## Discussion

Patients’ immune status may affect the treatment responses in various cancer therapies; however, the profiles of the TCR repertoire in baseline and follow-up CRC patients are still unclear. This is the first study to comprehensively profile the TCRα and TCRβ repertoires in 103 samples, including treatment-naïve and approximately 16-month follow-up CRC subjects, using next-generation sequencing. Our study demonstrated that the dynamic change in TCRα and TCRβ diversity was correlated with the levels of tumor markers (CEA and CA19-9) and tumor size after medical treatment, which demonstrated the prognostic potential of the TCR repertoire for monitoring treatment responses in CRC. Furthermore, TCRα dynamics analysis showed a more effective approach for distinguishing patient treatment response than TCRβ variation in our cohort. These findings provide solid methodology and observations of decreased TCR diversity correlating with response to chemotherapy in mCRC and an indication of correlation with response to therapy, and the noninvasive repertoire profiling using peripheral blood provides useful clinical information and expands the understanding of oncoimmunology.

How TCR diversity impacts effective adaptive immunity in cancer patients remains unclear. Our current study demonstrated that patients with higher TCRβ diversity at baseline had better progression-free survival than those with lower TCRβ diversity. The immune repertoires in healthy individuals are highly diverse and contribute to the selection of useful antigen-specific T cells under disease conditions. Higher TCRβ diversity at baseline may help immune activation against specific tumor antigens in patients. Thus, baseline TCRβ diversity indicates the adequate parameter of tumor immune surveillance and might be a promising factor for predicting prognoses, and peripheral specimen analysis appears a non-invasive monitoring approach for mCRC patients who are surgical inoperable. However, peripheral blood modulations may or may not correlate with intratumoral TCR diversity. The analysis on intratumoral TCR clonotypes may strengthen the notion that such clonotypes may be tumor antigen-specific and associated with response to therapy, and which provides a mechanistic rationale for the indicated correlation of decreased diversity and response to therapy.

According to a previous report, decreased TCRα and TCRβ repertoire diversity was present in elderly people compared with younger subjects; these phenomena were also observed in our healthy controls. No correlation between age and TCR repertoire diversity was observed, suggesting disturbed TCR repertoires in CRC patients. CDR3 is the most variable and directly determines the antigen-binding specificity of TCR. Some neoantigens generated by mutations in tumors may contribute to the generation of specific CDR3 clonotypes. The J32 segment of TCRα (TRAJ32) was significantly enhanced in CRC-pre patients compared to healthy controls (**Figure 3A**). Tumor cell can be eliminated *via* tumor antigen-specific T cells by targeting the peptide/major histocompatibility complex (MHC) derived from tumor-associated antigens. The result may suggest that specific chimeric T cell clonotype may be used as a TCR-based immunotherapy in tumor. Owing to the individual differences and tumor heterogeneity, no specific clonotype was recognized in all CRC patients; some specific CDR3 sequences were exclusively expressed in at least 25% of CRC patients (4 out of 16 patients) but not in healthy controls (**Supplementary Tables S2, S3**). It is very interesting to characterize whether these unique CDR3 clonotypes can be detected in the corresponding tumor microenvironment in the future. These may be neoantigen-specific T cells and might be good targets for adoptive cell therapies, and which may serve as bio-signature for distinguishing therapeutic response and/or tumor expansion. Sherwood et al. reported that some specific TCRβ CDR3 clonotypes were detected in CRC tumor tissues (33). However, potential public TCR repertoires specific to CRC patients in peripheral blood or tumors still require a larger sample size for characterization. We accessed the power of sample size in **Figure 5C**, and the power is 0.5. If the case and control ratio are 1:1, it needs sample size = 65 to reach the power = 0.8. Despite the limitation of sample size in this study, our results still gave a new insight that TCR repertoire sequencing could be a valuable approach for evaluating treatment response.

The coefficient analyses of TCRα and TCRβ VJ usage and CDR3 species showed the unique gene configuration of each subject and a higher association between paired follow-up samples than between different individual subjects. Compared to baseline TCRα diversity, CRC012, CRC013, CRC016, and CRC017 patients were categorized into the ΔChao1 > 0 group and had poor treatment response (SD/PD) after chemotherapy (**Supplementary Table S1**), and no apparent TCR repertoire variation in the current analysis was observed. Additionally, there was a trend of similar CDR3 peptide length distribution in these four patient types during therapy, and this result moderately supported that no apparent TCR repertoires were enriched in patients with poor treatment responses (**Figure 6C** and **Supplementary Figure S7**). When compared to baseline TCRα diversity, CRC001, CRC002, CRC010, and CRC015 patients were categorized into the ΔChao1 < 0 group after therapy and had a better treatment response (PR) at every chemotherapy time point (**Supplementary Table S1**).

With advances in sequencing technology, directly sequencing the TCRα and TCRβ gene loci can capture the true diversity of the TCR repertoires. Our current work performed a multiplex PCR assay and high-throughput sequencing in CRC longitudinal cohorts to improve the understanding of the dynamic immune responses during cancer therapy and found that a decreased Chao1 index was correlated with declining CEA and CA19-9 biomarker levels instead of other parameters, including sex, age, and hematological characteristics. Furthermore, TCR diversity was successful in distinguishing therapeutic responses. Because the immune repertoire profiling between cancer patients at the initial time point presents great heterogeneity. TCR diversity is generated by recombination, random insertion/deletion of gene segments of the TCR in the thymus; the TCR diversity has the potential to create around 10^15^ ~ 10^20^ TCR clonotypes (34, 35). Also, the relative amount of each TCR clonotype in a patient was relatively lower among the whole T cell population. The sample size in the current study is limited and we still need to enroll another large independent cohort to explore the significance of individual T cell clontype for predicting disease survival or treatment response.

The current study aims to provide the global evaluation of the dynamic changes of TCR colontypes during the course of chemotherapy. Indeed, we can’t know where the TCR clonotypes come from either CD8 T cells or CD4 T cells. If the overall TCR diversity can serve as a biomarker for predicating the disease survival or cancer treatment responses, the utility of TCR biomarker will be easier and simpler for clinical practice. In the future, using the purified CD4 T or CD8 T cells from patients for TCR sequencing can further investigate the possibility regulatory mechanisms for each TCR colontypes in cancer. TCR diversity analysis still needs to be improved for accuracy and practice in a larger CRC cohort.

Our data showed that the number of lymphocyte or monocyte may be associated with the changes of TCR diversity during chemotherapy. The lymphocyte to monocyte ratio may serve as a prognostic marker in stage III colon cancer. Furthermore, changes in neutrophil/lymphocyte and platelet/lymphocyte ratios after chemotherapy correlate with treatment response and prediction of prognosis in patients with gastric cancer. Growing evidence suggests an important role of immune cell population in cancer treatment responses. In the future, we can enroll a large independent cohort of metastatic colon cancer to further investigate this issue. Collectively, our findings demonstrate the importance and significance of immune status in cancer treatment.

## Data Availability Statement

The original contributions presented in the study are publicly available. This data can be found here: https://www.ncbi.nlm.nih.gov/geo/query/acc.cgi?acc=GSE182031.

## Ethics Statement

The studies involving human participants were reviewed and approved by Institutional Review Board at Chang Gung Memorial Hospital, Taiwan. The patients/participants provided their written informed consent to participate in this study.

## Author Contributions

Conception and design, H-CH, HL, and C-YY. Development of methodology, Y-SL, HL, and C-YY. Acquisition of data, H-CH, HL, C-YC, and C-YY. Analysis and interpretation of data (e.g., statistical analysis, biostatistics, computational analysis), Y-TC, Y-SL, and C-YY. Writing, review, and/or revision of the manuscript, Y-TC, H-CH, and C-YY. Administrative, technical, or material support, Y-TC, H-CH, Y-SL, and BT. Study supervision, C-YY. All authors contributed to the article and approved the submitted version.

## Funding

This work was supported by grants from the Ministry of Science and Technology of Taiwan (107-2314-B-182-075-MY3, 110-2314-B-182-046 to C-YY) and Chang Gung Memorial Hospital (CMRPG3I0311 and CMRPG3G2071 to H-CH, CMRPD1K0402 to C-YY). This work was also financially supported by the “Research Center for Emerging Viral Infections” and the “Molecular Medicine Research Center” from the Featured Areas Research Center Program within the framework of the Higher Education Sprout Project by the Ministry of Education (MOE) in Taiwan and the Ministry of Science and Technology (MOST), Taiwan (MOST108-3017-F-182-001). NGS experiments and bioinformatics analyses were performed at the Genomics NGS Laboratory Molecular Medicine Research Center, Chang Gung University, Taiwan (EMRPD1L0281, CLRPD1J0013).

## Conflict of Interest

The authors declare that the research was conducted in the absence of any commercial or financial relationships that could be construed as a potential conflict of interest.

## Publisher’s Note

All claims expressed in this article are solely those of the authors and do not necessarily represent those of their affiliated organizations, or those of the publisher, the editors and the reviewers. Any product that may be evaluated in this article, or claim that may be made by its manufacturer, is not guaranteed or endorsed by the publisher.
