# Supplementary material for: Longitudinal High-Throughput Sequencing of the T-Cell Receptor Repertoire Reveals Dynamic Change and Prognostic Significance of Peripheral Blood TCR Diversity in Metastatic Colorectal Cancer During Chemotherapy
Source: Front Immunol. 2022 Jan 12;12:743448. doi: 10.3389/fimmu.2021.743448 (PMC8789675; doi:10.3389/fimmu.2021.743448)
Supplement: Supplementary file 1 [file Presentation_1.pdf]

Supplementary information

Longitudinal high-throughput sequencing of the T-cell receptor repertoire reveals dynamic change and prognostic significance of peripheral blood TCR diversity in metastatic colorectal cancer during chemotherapy

Yi-Tung Chen[1,2]#, Hung-Chih Hsu[3,4]#, Yun-Shien Lee[5], Hsuan Liu[1,6,7,8], Bertrand Chin-Ming Tan[2,7,9,10], Chia-Yin Chin[1], Ian Yi-Feng Chang[1,10], Chia-Yu Yang[1,7,11,12]*

[1]Molecular Medicine Research Center, Chang Gung University, Taoyuan, Taiwan
[2]Research Center for Emerging Viral Infections, Chang Gung University, Taoyuan, Taiwan
[3]Division of Hematology-Oncology, Chang Gung Memorial Hospital at Linkou, Tao-Yuan, Taiwan
[4]College of Medicine, Chang Gung University, Tao-Yuan, Taiwan
[5]Department of Biotechnology, Ming Chuan University, Taoyuan 333, Taiwan
[6]Department of Cell and Molecular Biology, College of Medicine, Chang Gung University, Taoyuan, Taiwan
[7]Graduate Institute of Biomedical Sciences, College of Medicine, Chang Gung University, Taoyuan, Taiwan
[8]Division of Colon and Rectal Surgery, Chang Gung Memorial Hospital, Taoyuan, Taiwan
[9]Department of Biomedical Sciences, College of Medicine, Chang Gung University, Taoyuan, Taiwan
[10]Department of Neurosurgery, Lin-Kou Medical Center, Chang Gung Memorial Hospital, Taoyuan, Taiwan
[11]Department of Microbiology and Immunology, College of Medicine, Chang Gung University, Taoyuan, Taiwan
[12]Department of Otolaryngology-Head and Neck Surgery, Chang Gung Memorial Hospital, Taoyuan, Taiwan

[#]These authors contributed equally to this work.
*Correspondence Author: Chia-Yu Yang, Department of Microbiology and Immunology, College of Medicine, Chang Gung University, No.259, Wenhua 1st Rd.,

Guishan Dist., Taoyuan City 33302, Taiwan (R.O.C.) E-mail:
chiayu-yang@mail.cgu.edu.tw

Running title: Prognostic significance of TCR diversity in metastatic CRC

Keywords: T-cell repertoire, metastatic colorectal cancer, prognosis, peripheral blood, chemotherapy

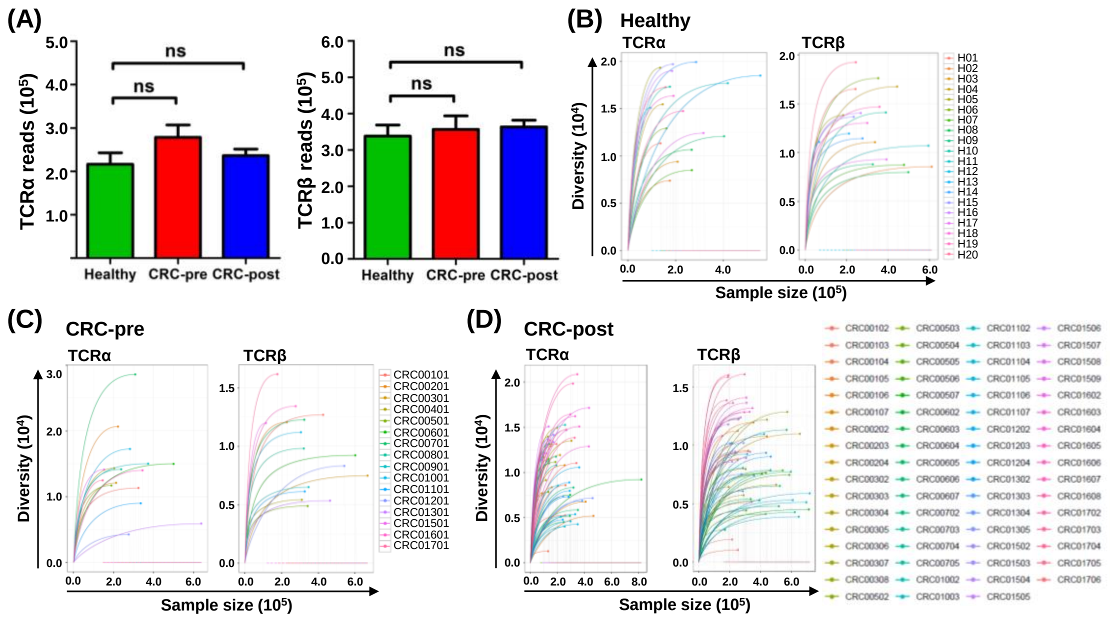

Supplementary Figure S1. The sequencing read coverage for the TCRα and TCRβ repertoires.

(A) The number of TCRα and TCRβ sequencing read counts of healthy controls (green column), CRC-pre (red column), and CRC-post (blue column) subjects in this study are presented as bar graphs. Statistical significance was measured using Student's *t*-test. (B) Rarefaction analysis of TCRα and TCRβ repertoires from healthy controls (H1 to H2O, H20, n = 20) was performed, and the diversity (y-axis) of each sample against its sample size (x-axis) was plotted. (C) Rarefaction plots of the TCRα and TCRβ repertoires from CRC-pre subjects were generated, and a total of 16 samples (CRC00101 to CRC01701) were analyzed. (D) Rarefaction analysis of the TCRα and TCRβ repertoires from CRC-post subject were performed, a total of 83 samples (CRC00102 to CRC01706) were assayed.

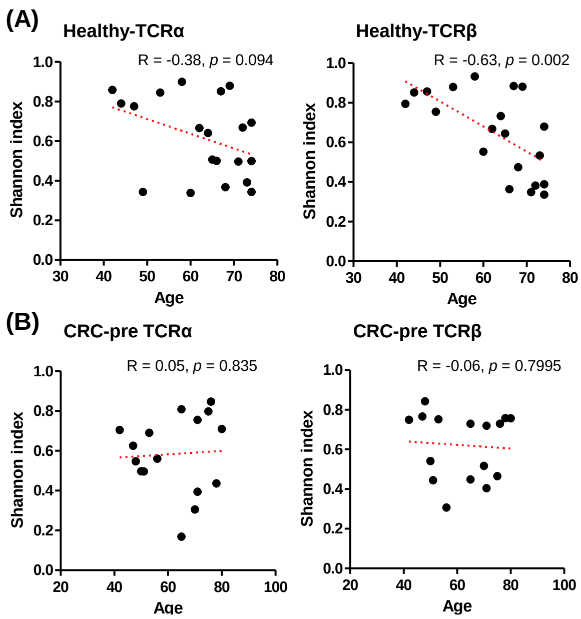

Supplementary Figure S2. Correlation between TCRα and TCRβ repertoire diversity index and age in healthy controls and CRC patients.

Pearson correlation analysis between age (years) and Shannon diversity of TCRα and TCRβ repertoires in healthy controls (A) and CRC patients (B) was performed, and the red dotted line indicates the regression model. The Pearson r value (R) and statistical *p*-value are shown.

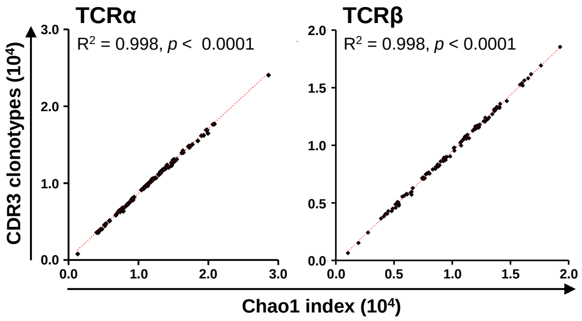

Supplementary Figure S3. The Chao1 diversity index was positively correlated with CDR3 clonotypes.

Linear regression analysis between the Chao1 diversity index (x-axis) and CDR3 clonotypes (y-axis) from the TCRα and TCRβ repertoires in all samples (n = 103) was performed, and the obtained R square and *p*-value are indicated in the upper panel.

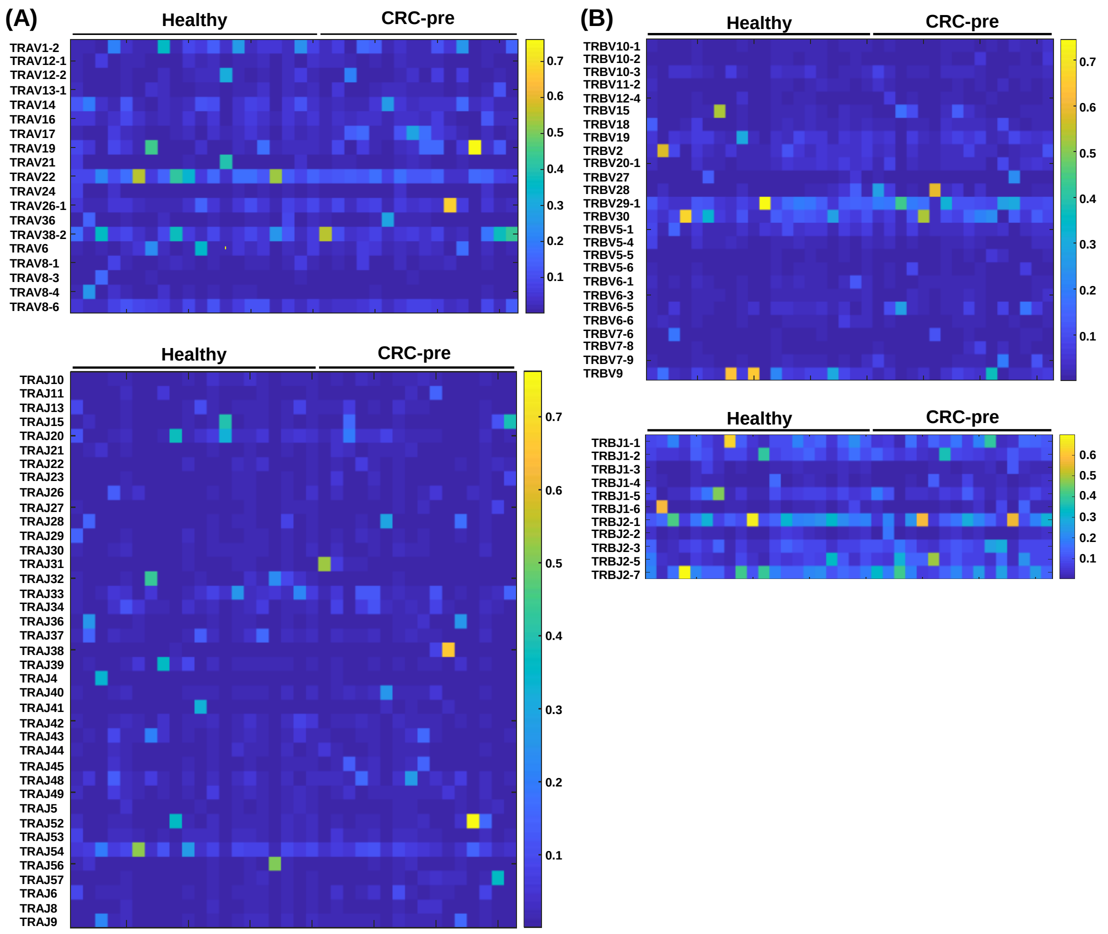

Supplementary Figure S4. Heatmap of the overall usage of TCRα and TCRβ V-J gene segments in healthy controls and CRC patients.

(A) The heatmap displays the frequency of the TRAV (upper panel) and TRAJ genes (lower panel) in healthy and CRC-pre patients, and the expression scale bar is shown on the right. (B) Heatmap representation for the frequency of the TRBV (upper panel) and TRBJ genes (lower panel) from healthy and CRC-pre patients. The corresponding expression scale bar is displayed on the right.

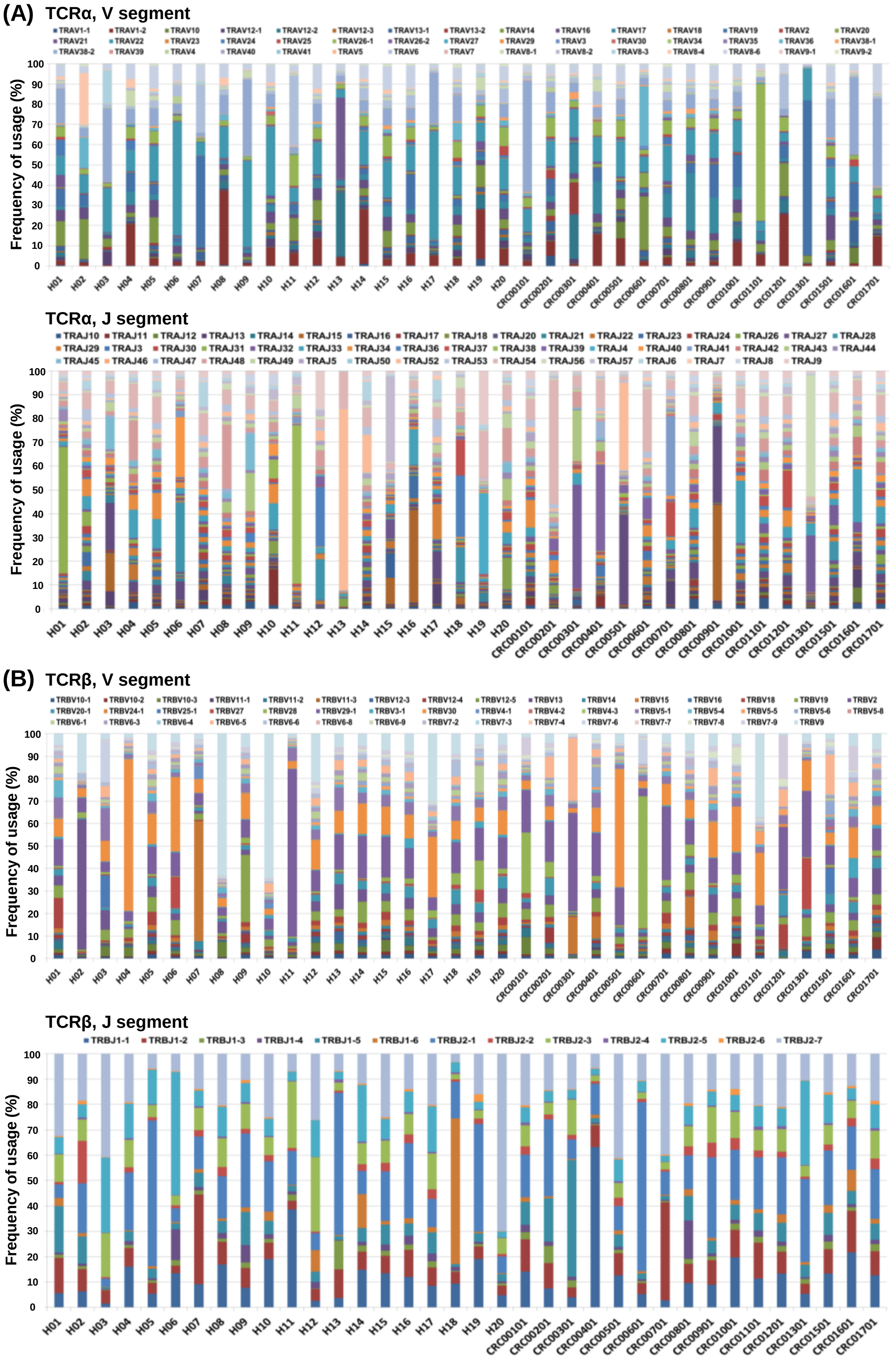

Supplementary Figure S5. Frequency of TCRα and TCRβ V and J gene usage in healthy and CRC patients.

(A) Expressed TCRα variable genes (TRAV, upper panel) and TCRα joining genes

(TRAJ, lower panel) from healthy controls (H01 to H20 and CRC-pre patients (CRC00101 to CRC01701) were color coded and illustrated as stacked bar graphs, and the expression frequency of each segment is shown in percentages. (B) The expressed frequencies of TCRβ variable genes (TRBV) and joining genes (TRBJ) from healthy controls and CRC-pre patients are described in a color-coded stacked percentage bar chart.

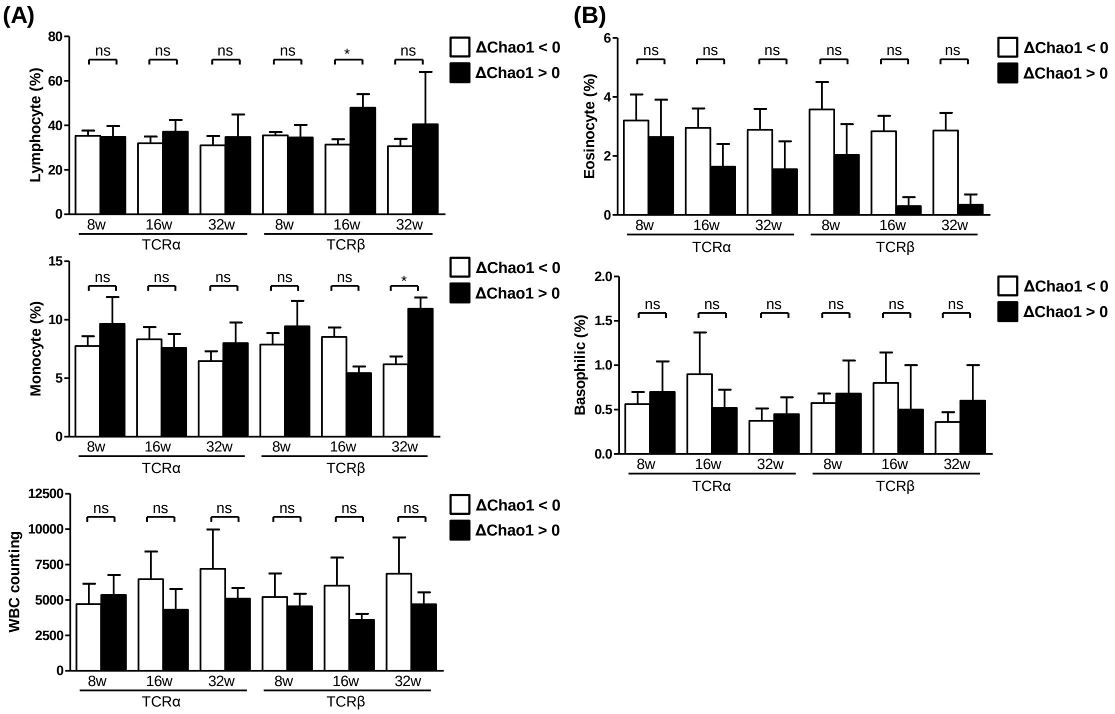

(C)

Variable	Hazard ratio	P-value	95% CI	
			Lower	Higher
TCRβ diversity	0.844	0.015	0.736	0.967
Tumor size	1.009	0.626	0.972	1.048
CEA value	1.001	0.322	0.999	1.002

Supplementary Figure S6. Statistical comparison of blood cell counts in follow-up CRC patients.

(A) CRC-post samples collected at 8 weeks, 16 weeks, and 32 weeks were divided into the Chao1 decreasing subgroup (ΔChao1 < 0, white columns) and the Chao1 increasing subgroup (ΔChao1 > 0, black columns) based on Chao1 deviation from TCRα and TCRβ repertoire profiling. The lymphocyte percentage, monocyte ratio, and white blood cell (WBC) counts of the Chao1 subgroups are shown in bar graphs, and statistical significance was determined by unpaired Student's t-test. (B) Eosinophil and basophil percentages in the Chao1 subgroups as described in A are displayed in bar graphs, and statistical significance was assessed using unpaired Student's t-test. (C) The COX regression analyses for estimating the potential risk factors for predication progression-free survival with the indicated variance, including TCRβ diversity, tumor size, and CEA value.

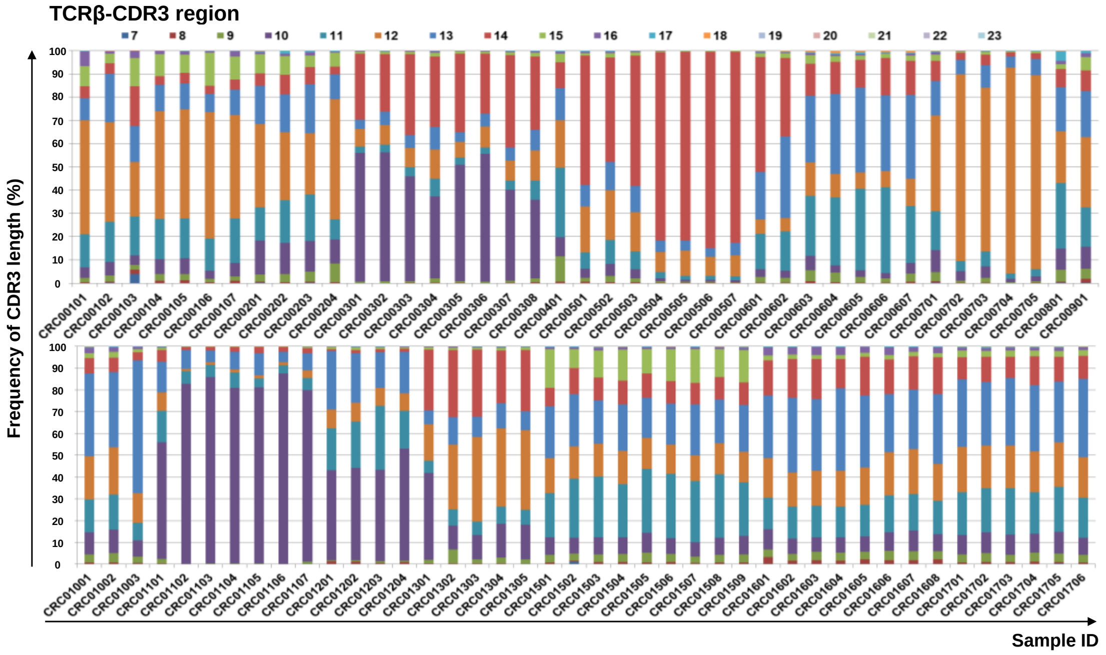

Supplementary Figure S7. CDR3 coding length in TCRβ repertoires in follow-up patients.

CDR3-coding amino acid length and frequency of CRC-pre and CRC-post TCRβ repertoires are illustrated as color-coded percentage bar graphs. The sample ID is the x-axis, and the amino acid length frequency is the y-axis.

(A) **Pearson correlation for TCRα-V gene**

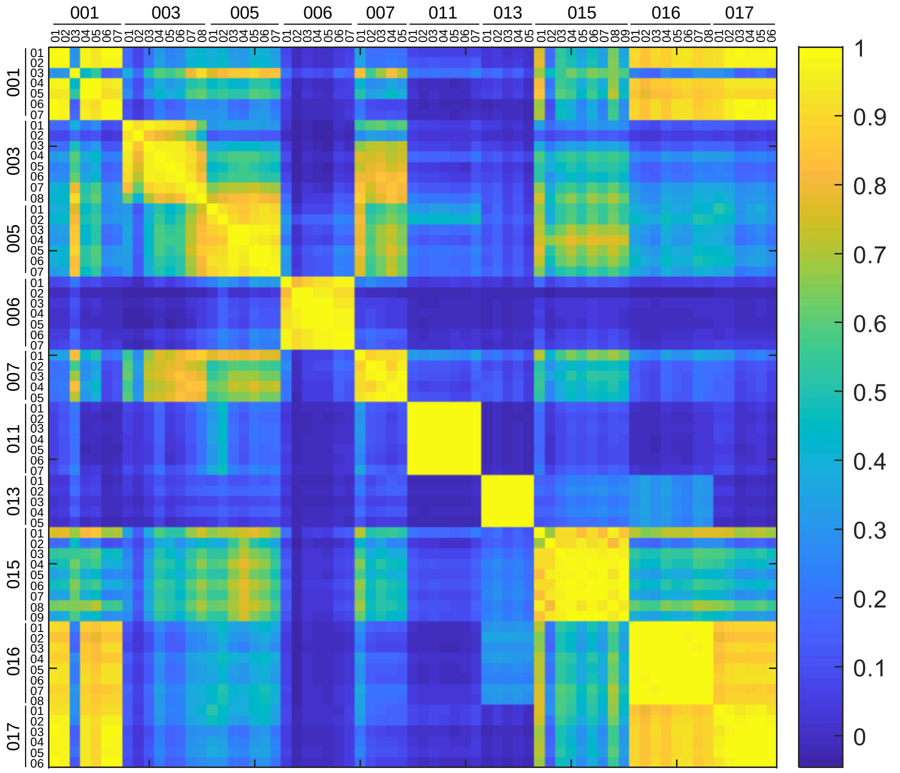

Pearson correlation for TCRα-J gene

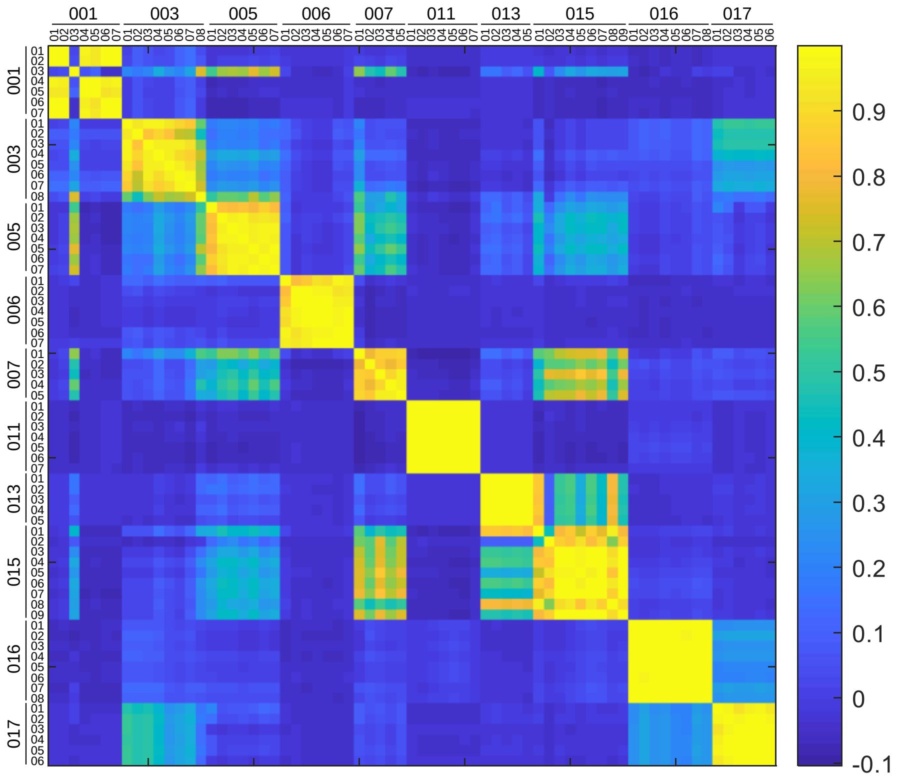

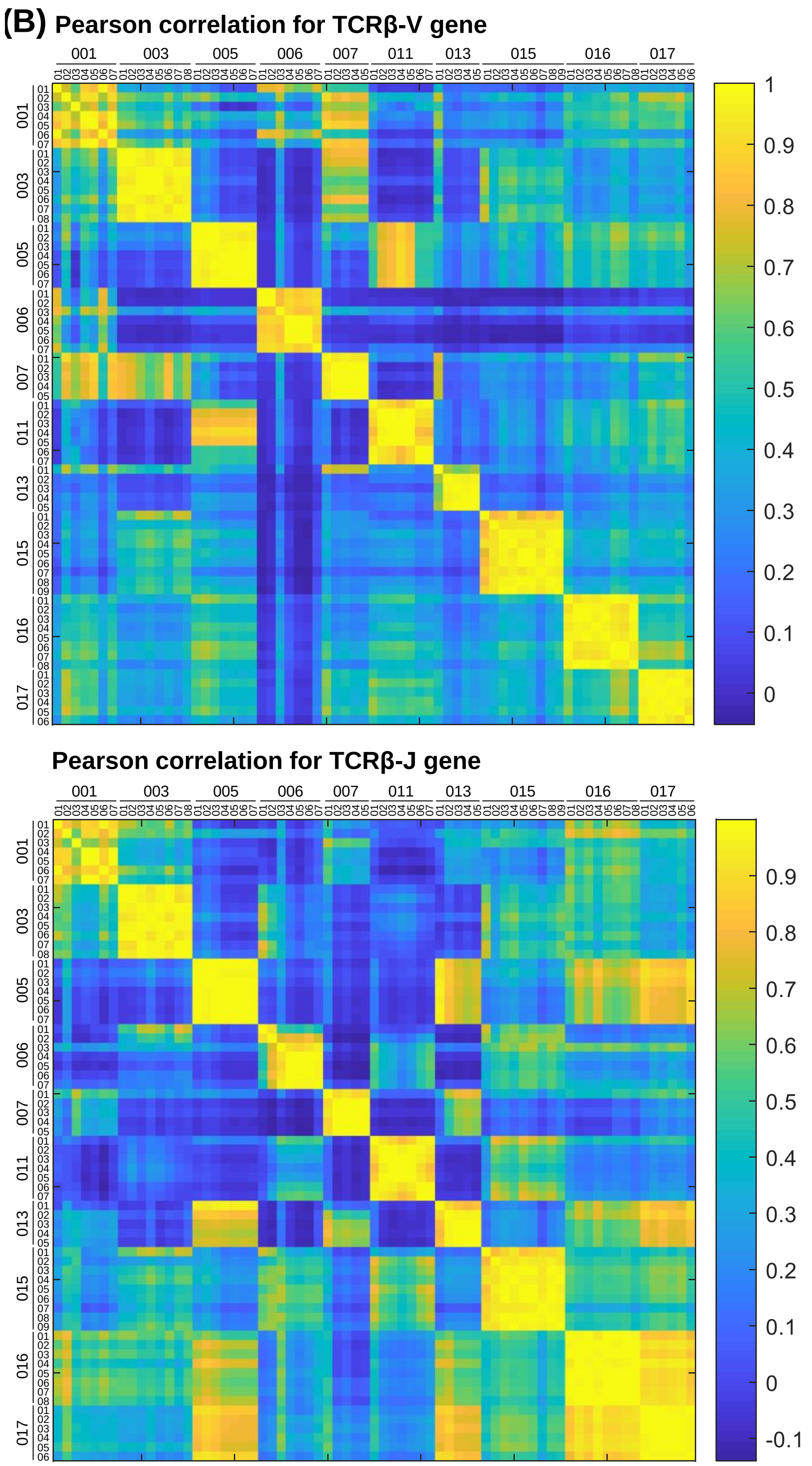

Supplementary Figure S8. Correlation analyses of TCR repertoires in follow-up patients.

Pearson correlation analysis between expressed TCRα (A) and TCRβ (B) variable genes (V) and joining genes (J) of the follow-up CRC samples was performed, and the results are shown in a heat map. The correlation coefficient between any two given samples (x-axis versus y-axis) was colored coded based on the aside coefficient scale bar. The coefficient scale bar is shown in the right panel.
